# Dr. Edward W. Bryan

**Published:** 1918-10

**Authors:** 


**Dr. Edward W. Bryan** of Corning died August 24th, age 85. He graduated from the Cleveland Medical School in 1873.

